# Editorial: The Dynamics of Stress Granules

**DOI:** 10.3389/fcell.2021.789678

**Published:** 2021-11-16

**Authors:** Subhash Mehto, Beidong Liu, Ronggui Hu, Santosh Chauhan

**Affiliations:** ^1^Cell Biology and Infectious Diseases Unit, Institute of Life Sciences, Bhubaneswar, India; ^2^Department of Chemistry and Molecular Biology, University of Gothenburg, Göteborg, Sweden; ^3^Faculty of Science, Center for Large-Scale Cell-Based Screening, University of Gothenburg, Göteborg, Sweden; ^4^State Key Laboratory of Subtropical Silviculture, School of Forestry and Biotechnology, Zhejiang A&F University, Hangzhou, China; ^5^Cancer Center, Shanghai Tenth People's Hospital, School of Medicine, Tongji University, Shanghai, China; ^6^State Key Laboratory of Molecular Biology, Shanghai Institute of Biochemistry and Cell Biology, Center for Excellence in Molecular Cell Science, Chinese Academy of Sciences, Shanghai, China

**Keywords:** stress granules, RNA binding protein, neurodegeneration, stress response, post transcriptional gene regulation, lipid droplets

Stress granules (SGs) are heterogeneous cytoplasmic structures of phase-separated RNA and proteins that are formed when cells are exposed to environmental or intrinsic stresses including viral infection, temperature change, or oxidative stress. Under these conditions, mRNA translation is inhibited and cells sequester mRNA, RNA-binding proteins (RBPs), and several other components of protein translational machinery into the SGs. The formation of SGs is a protective cell response and in most physiological conditions helps the cells to cope with the stress or furnish a hostile environment for viral invasion. However, in certain conditions such as cancer and chemoresistance, the SGs provide increased fitness and enhanced capability to survive in harsh conditions. Understanding of this membrane-less organelle and its function has increased significantly in recent times. Here, eight articles published on this Research Topic, are elucidating the roles and function of SGs in stressed cells and diseases.

The protein composition of SGs and the function of these proteins in SGs dynamics has been well-studied over the last decade. New studies suggest the role of RNA in SGs dynamics. Campos-Melo et al. scrutinized the existing evidence in support of RNA playing an important role in the formation of SGs and also reviewed the concept of SGs as therapeutic targets. In addition to the emerging role of RNA in SGs dynamics, there is increasing interest in understanding the role of post-translational protein modifications in SG organization. In this context, Jin et al. reviewed the latest advances on the roles of poly(ADP-ribose) in the regulation of SG formation and dynamics. The activation of immune cells leads to transcriptional, post-transcription, and translational programs for inducing differentiation or proliferation that are essential for an efficient immune response. Curdy et al. reviewed the current knowledge about the role of stress granules in post-transcriptional regulation in immune cells.

Stress granules formation is connected to several diseases, including neurodegeneration. The formation of SGs is considered a pro-survival strategy in neurons to ease out the intermittent stresses. However, persistent stress and mutation in RNA binding proteins could increase SGs in the neuron that could be toxic leading to pathology. Jeon and Lee reviewed the advances in this field and also provide insight for potential therapeutic approaches to resolve the persistent SGs associated with neurodegeneration. In understanding the role of SGs in neurodegeneration, Ding et al. article presented an interesting work in the context of the role of RBP TDP-43 in amyotrophic lateral sclerosis (ALS). The studied dynamics of wild type and mutant TDP-43 in different stress conditions. They observed differential localization of mutant vs. wild type TDP-43 in stress granules during stress where the mutant TDP-43 was present in SGs compared to the presence of wild type in the nucleus. The data suggest the role of mutant TDP-43 in the dynamics of SGs and the seeding of insoluble cytoplasmic inclusions observed in ALS.

Nowee et al. studied the interaction between Alphavirus proteins and SGs. Specifically, they studied the role of the non-structural protein 3 (nsP3) hypervariable domain that can interact with several host proteins associated with SGs. To better understand nsP3-host protein interactions, they performed exhaustive co-localization experiments with the nsP3s of 20 diverse alphaviruses with SG proteins including G3BP, FMRP, and BIN1.

Tellurium oxyanion, tellurite (TeO3–2), is a highly toxic industrial compound and has been associated with adverse effects on human health. Gaete-Argel et al. found that tellurite induces the assembly of cytoplasmic as well as nuclear SGs in response to oxidative stress and DNA damage where the nuclear SGs were co-localized with DNA damage marker γH2AX.

In another interesting study presented in this issue, Amen and Kaganovich demonstrated a novel connection between the biogenesis of SGs and lipid droplets through a small-molecule screen. Interestingly, they found that enhancing of SGs in cells leads to the formation of lipid droplets, and failure to induce lipid droplet response results in failure of SGs biogenesis suggesting that common regulatory pathways are involved in both stress responses.

Altogether, this issue on stress granules reviews the extensive knowledge in the regulation of the biogenesis of SGs and their role in diseases. Also, provides new knowledge in their function in viral infection and stress responses.

## Author Contributions

All authors listed have made a substantial, direct and intellectual contribution to the work, and approved it for publication.

## Conflict of Interest

The authors declare that the research was conducted in the absence of any commercial or financial relationships that could be construed as a potential conflict of interest.

## Publisher's Note

All claims expressed in this article are solely those of the authors and do not necessarily represent those of their affiliated organizations, or those of the publisher, the editors and the reviewers. Any product that may be evaluated in this article, or claim that may be made by its manufacturer, is not guaranteed or endorsed by the publisher.

